# The acceptability and perceived use of HIV self-testing among technical vocational education and training students in Limpopo province

**DOI:** 10.4102/hsag.v28i0.2095

**Published:** 2023-03-07

**Authors:** Mimi E. Teffo, Samuel L. Mndzebele, Mathildah M. Mokgatle

**Affiliations:** 1 Department of Epidemiology and Biostatistics, School of Public Health, Sefako Makgatho Health Sciences University, Ga-Rankuwa, South Africa; 2 Department of Epidemiology and Biostatistics, School of Public Health, Sefako Makgatho Health Sciences University, Ga-Rankuwa, South Africa; 3 Faculty of Education, Institute of Development Management, Mbabane, Eswatini

**Keywords:** sexual behaviour, sexual partners, young people, HIV testing, condom use

## Abstract

**Background:**

Human immunodeficiency virus self-testing (HIVST) is a most recent testing modality to reach young people to test for HIV, due to their increased vulnerability of contracting HIV. Limited literature is available describing sexual behaviours and the acceptability of HIVST and its perceived use among students.

**Aim:**

The aim of this study was to assess the acceptability and perceived use of HIV self-testing among students in Limpopo province, South Africa.

**Setting:**

The study was conducted in Limpopo province, at a technical and vocational education and training (TVET) college.

**Methods:**

A cross-sectional study was conducted with a sample of 396 students recruited from a TVET college.

**Results:**

The mean age of the students was 22.9 years, with the majority of the students being female (77.2%). The majority (81.4%) of the students sampled reported regular sexual activity. Sixty per cent of the students had used condoms during their last sexual encounter. The acceptability of HIVST was high, with more women showing the willingness to take up HIVST (82.5%). Being sexually active (odds ratio [OR] 1.23; (confidence interval [CI]: 2.14 -6.94; *p* = 0.000), a number of sexual partners (OR 1.045; CI: 1.98 -10.02; *p* = 0.000) and condom use during the last sexual encounter (OR 0.62; CI: 3.81 -9.59; *p* = 0.000) were factors associated with HIVST.

**Conclusion:**

The high acceptability of HIV shows a need for innovative demand creation in sexual and reproductive health (SRH) programming.

**Contribution:**

The study contributes to the body of literature about the acceptability and perceived use of HIV self-testing among students. Findings can be used for improving HIVST interventions using innovative approaches.

## Introduction

Globally, numerous countries have developed human immunodeficiency virus self-testing (HIVST) policies, and implementation is growing rapidly, according to the World Health Organization (WHO 2019). Worldwide, 59 countries have taken up HIVST, and a further 53 are developing countries (WHO 2018). HIV self-testing is legal in 23 countries in Europe, with 14 countries having dedicated, national self-testing policies to support and roll out implementation (Integrate 2021). In sub-Saharan Africa, HIV testing is widely offered but remains delayed by the psychological burden of knowing one’s status and economic barriers (Musheke et al. 2013). Moreover, HIVST rapid kits are available for use, sale and distribution in other parts of Africa such as Kenya, Nigeria and South Africa (SA) (HIVST 2020). There are a number of initiatives testing the viability of a wide-scale distribution of these kits within other sub-Saharan countries (Smith et al. 2016). In SA, HIVST was included as a complementary strategy in the *National HIV Testing Services Policy* in 2016, and the HIVST guidelines were incorporated in SA’s *National Strategic Plan on HIV, Tuberculosis (TB) and Sexually Transmitted Infections (STIs)* 2017–2022 (Venter et al. 2017). Also, HIVST is available for use and secondary distribution in SA, although a confirmatory test needs to be conducted at a health facility, according to the HIVST algorithm.

HIV testing is viewed as an entry to HIV prevention, and SA has made significant efforts, including mass HIV testing campaigns, to achieve the United Nations’ (UN’s) ambitious 90–90–90 global HIV targets. Furthermore, SA has an increasingly young population, with young people constituting 37% of the country’s population (StatsSA 2014). This presents a great opportunity for the country and can serve as a powerful resource; however, HIV remains one of the biggest challenges for the youth in SA, particularly young women. Quite a few reasons have been cited for this, including, *inter alia*, risky sexual behaviour such as inconsistent condom use, multiple concurrent partnerships, early sexual debut (Eaton, Flisher & Aarø 2003; Menon et al. 2016) and social and structural drivers (Dellar, Dlamini & Karim 2015). A growing body of evidence indicates that the risk of acquiring HIV drives forward the vulnerability to a plethora of health risks such as STIs (Alemu et al. 2007; Argardh et al., 2012; Kamara et al. 2019; Menon et al. 2016; Nalwadda et al. 2010; Zuma et al. 2016). It is reported that out of every seven new infections worldwide in 2019, two were among young people between 15 and 24 years of age (UNAIDS 2021). In other studies (Dellar et al. 2015; Zuma et al. 2016), it was concluded that the risk of acquiring HIV also increases in the presence of STIs, with the majority of STIs occurring in young people below 25 years of age.

Comparable to their peers of the same age group, students in universities are at high risk of contracting HIV (Peltzer 2000). Previous studies have suggested a high rate of risky sexual behaviour among youth in institutions of higher learning in Africa. About 78% of the study participants at a rural South African university had experienced sexual encounters (Heeren et al. 2012). This is contrary to the findings at a peri-urban university in the Democratic Republic of Congo (DRC), of which 51.8% of the students reported being sexually active. A study among male and female university students in Pretoria, South Africa, showed that 39.3% of the sexually active students reported that they did not use condoms and 31.7% had concurrent sexual partners (Mokgatle, Madiba & Cele 2021). This figure is more similar to the findings of a university study in Ethiopia, whereby 30.14% of the students indicated that they were engaged in multiple concurrent sexual relationships. However, at a university in Zambia, the students in Year 4 who reported having more than one sexual partner significantly increased to 64% female and 50% male students (Menon et al. 2016).

The South African government has made HIV testing efforts to mobilise young people to access youth-friendly HIV testing services (Ramirez-Avilla et al. 2012). Despite these efforts, under-served key populations such as youth still do not have adequate access to HIV testing services (Makusha et al. 2015). However, a number of studies conducted among college and university students regarding HIVST have shown similar results in relation to HIVST. A study conducted in South Africa among student nurses in a private nursing college showed that most participants found HIVST to be acceptable (Madiba, Segobola & Mokgatle 2015). Similarly, a college study in Gauteng and North West provinces, South Africa, found that the acceptability of HIVST was high (81.7%), with three-quarters of students showing willingness to purchase a self-test kit. The acceptability of HIVST in the study was associated with being sexually active and having multiple sexual partners (Mokgatle & Madiba 2017). In a university in the DRC, the acceptability of HIVST by students was also found to be high (81.4%). Contrary to the study in Gauteng and North West provinces, South Africa, factors associated with HIVST uptake were knowledge of the importance of the test, perceptions of pre- and post-test counselling and the willingness to test with a partner.

## Methods

### Study design

A cross-sectional study design was employed to assess the sexual behaviours and acceptability and perceived use of HIVST among technical vocational education and training (TVET) students.

### Study setting

The study was conducted at Capricorn College for TVET at Polokwane Campus in Capricorn District in Limpopo province. The TVET college is situated in Polokwane city, a predominantly urban area of the province. Technical vocational education and training colleges are one of the institutions of higher learning in South Africa that provide technical vocational education with the aim of adequately preparing young people with the knowledge and artisan skills that are required in the labour market. Capricorn College hosts four campuses in the district; however, only one campus participated in the study, as the other three were difficult to reach. The majority of these students who attend the TVET come from rural areas and disadvantaged communities.

### Study population

The study participants were students in Level 2 – Level 4, enrolled for the following programmes: Tourism, Information and Communication Technology (ICT), Marketing, Office, Transport, Safety and Finance. Around 5600 students were registered in this campus at the time of data collection. Of these, 396 students were recruited to participate in the study using simple random sampling. The students attended morning and afternoon classes between Mondays and Fridays, and the data were collected during free classes to avoid disrupting teaching and learning time.

### Data collection

A presentation about the purpose of the study and selection criteria was presented to the campus manager and the head of department after permission was granted. A validated questionnaire was adapted from the Australian Secondary Students and South African Sexual Health Survey (Mokgatle & Madiba 2017; Smith 2009) as a method of data collection. Moreover, the tool was pretested to avoid ambiguous questions. The study participants who participated in pretesting of the tool were similar to the sample in the study population. The structured questionnaire was divided into two sections: students’ demographics and sexual behaviours and acceptability and perceived use of HIVST. The tool was written in English and self-administered in the classroom. The students were informed that participation in the study was voluntary, and the purpose of the study was explained to them. The students were reassured of confidentiality, and written informed consent forms were distributed to the students who agreed to participate in the study. Furthermore, the students received instructions on the completion of the questionnaire. The questionnaire did not gather any personal information of the students such as names and personal identifiers to ensure anonymity. The researcher was accompanied by the course facilitator in the classrooms to introduce the researcher to the students on the days of data collection, after reporting to the college management. The course facilitator was neither in the classrooms when the researcher explained the purpose of the study nor during the data collection process. All of the college students in every class that was randomly selected were invited to participate on the day of data collection.

### Data analysis

The questionnaires were coded, and data were captured into Excel (Microsoft Corporation, Redmond, Washington, United States) and the statistical package Stata version 13 (StataCorp LLC, College Station, Texas, United States) was used for data analysis. The categorical variables such as gender and the level of acceptability were analysed using frequency tables. Bivariate analysis was conducted in order to assess an association and measure the level of significance. For logistic regression, only statistically significant variables were included from the bivariate analysis to limit the number of variables.

### Ethical considerations

The study was conducted after obtaining ethical approval from the Sefako Makgatho Health Sciences University Research Ethics Committee (ref. no. SMUREC/H/214/2017: PG) and permission from Capricorn College for TVET Central Office and the college management from the TVET College Polokwane Campus. The students were recruited after permission was sought from the college executive management. The participation in the study was voluntary, and consent was obtained from every student prior to commencement of the study. There were no personal identifications recorded on the questionnaire to ensure anonymity.

## Results

### Description of the participants

The demographics of the participants are described in Table 1. The mean age of the participants was 22.9 years (standard deviation [SD]: 2.7, age range 18–34 years), with 68.6% between the ages of 20 and 24 years. The participants in the study were predominantly female (77.2%). The participants were from three levels of study, of whom 45.5% were in Year 3 of training, followed by 27.9% in Year 2, while 26.6% were in Year 4. Most (74.9%) of them were living in rural areas, 22.6% were living in urban areas and 2.6% lived in informal settlements. The majority (87.9%) spoke Sepedi, 5.3% spoke Xitsonga, followed by Tshivenda (4.5%), while 2.4% spoke other languages.

**TABLE 1:** Characteristics of the study participants (*n* = 396).

Variables	Categories	Frequency	%
**Gender (missing values = 6)**			
	Male	89	22.8
	Female	301	77.2
**Age category (missing values = 7)**			
	0–19 years	24	5.9
	20–24 years	267	68.6
	25–29 years	89	23.1
	Above 30 years	9	2.3
**Home language (missing values = 16)**			
	Sepedi	334	87.9
	Xitsonga	20	5.3
	Tshivenda	17	4.5
	Other languages	9	2.4
**Areas of residence (missing values = 6)**			
	Rural areas	292	74.9
	Urban areas	88	22.6
	Informal settlements	10	2.6
**Level of study (missing values = 16)**			
	Level 2	106	27.9
	Level 3	173	45.5
	Level 4	101	26.6

*Source:* Mokgatle, M. & Madiba, S., 2017, ‘High acceptability of HIV self-testing among technical vocational education and training college students in Gauteng and North West Province: What are the implications for the scale up in South Africa?’, *PLoS One* 12(1), 1–14. https://doi.org/10.1371/journal.pone.0169765

### Sexual behaviour and testing practices of technical vocational education and training students

Most of the students (85.8%) had a sexual partner, and 81.4% reported regular sexual activity (Table 2). More than half (63.7%) reported having only one sexual partner, 18.7% two sexual partners and 17.6% more than two partners. Only 60.0% of the students reported the use of condoms during the last sexual act. A total of 11.6% of the students had never been tested for HIV, while 60.4% of the participants reported that they knew the HIV status of their sexual partner. The majority of the study participants, 76.0%, indicated their likelihood to discuss HIV testing with partner, and 81.0% said that they were likely to ask partners to go for HIV testing.

**TABLE 2:** Sexual behaviours and testing practices of technical vocational education and training students.

Variables	Frequency (*N*)	%
**Have a sexual partner (missing values = 1)**		
Yes	339	85.8
No	56	14.2
**Sexually active (missing values = 20)**		
Yes	306	81.4
No	70	18.6
**Number of sexual partners in the past year (missing values = 8)**		
One partner	239	63.7
Two partners	70	18.7
More than two partners	66	17.6
**Use condom first time (missing values = 4)**		
Yes	332	87.4
No	48	12.6
**Condom use during last sexual act (missing values = 9)**		
Yes	225	60.0
No	150	40.0
**Ever been tested for HIV (no missing values)**		
Yes	350	88.4
No	46	11.6
**Know HIV status of sexual partner (missing values = 2)**		
Yes	238	60.4
No	128	32.5
No partner	28	7.1
**Likely to discuss HIV testing with partner (missing values = 4)**		
Likely	297	76.0
Unlikely	64	16.4
Never	30	7.7
**Likely to ask partner to go for HIV testing (missing values = 6)**		
Likely	315	81.0
Unlikely	49	12.6
Never	25	6.4

*Source:* Mokgatle, M. & Madiba, S., 2017, ‘High acceptability of HIV self-testing among technical vocational education and training college students in Gauteng and North West Province: What are the implications for the scale up in South Africa?’, *PLoS One* 12(1), 1–14. https://doi.org/10.1371/journal.pone.0169765

Table 3 shows that there were significance gender differences with regard to willingness to purchase HIVST kits among the students, with more female students showing a willingness to buy HIVST kits versus male students (*p* = 0.03). The results further showed that there were no differences between male and female students for use of HIVST (*p* = 0.59), acceptability of HIVST (*p* = 0.45), willingness to submit HIVST results for health statistics (*p* = 0.90), willingness to confirm HIVST results at a health facility (*p* = 0.15) and willingness to test with a partner using HIVST kits (*p* = 0.18).

**TABLE 3:** Acceptability and use of HIV self-testing and counselling by gender.

Variables	Missing values	Total	Female		Male		*p*
			*n*	%	*n*	%	
**Ever heard of HIVST before (*N* = 390)**							
Yes	6	195	146	48.5	49	55.1	0.278
No		195	155	51.5	40	44.9	-
**Ever used HIVST before (*N* = 380)**							
Yes	16	42	31	10.6	11	12.6	0.590
No		338	262	89.4	76	87.4	-
**Willing to take up HIVST (*N* = 381)**							
Yes	15	311	242	82.5	69	78.4	0.454
No		46	35	12.0	11	12.5	-
Not sure		24	16	5.5	8	9.1	-
**Willing to buy HIVST kit (*N* = 390)**							
Yes	6	255	196	65.1	59	66.3	0.037
No		73	63	20.9	10	11.2	-
Not sure		62	42	14.0	20	22.5	-
**Pretest counselling is necessary for HIVST (*N* = 390)**							
Yes	6	236	179	59.5	57	64.0	0.337
No		97	80	26.5	17	19.1	-
Not sure		57	42	14.0	15	16.9	-
**Post-test counselling is necessary for HIVST (*N* = 384)**							
Yes	12	227	174	59.0	53	59.6	0.951
No		82	64	21.7	18	20.2	-
Not sure		75	57	19.3	18	20.2	-
**Willing to submit HIVST results for health statistics (*N* = 381)**							
Yes	15	253	196	66.9	57	64.8	0.908
No		72	54	18.4	18	20.4	-
Not sure		56	43	14.7	13	14.8	-
**Willing to confirm HIVST results at a health facility (*N* = 388)**							
Yes	8	301	236	78.9	65	73.0	0.158
No		54	42	14.1	12	13.5	-
Not sure		33	21	7.0	-	13.5	-
**Telephone hotlines sufficient for post-test counselling (*N* = 388)**							
Agree	8	176	136	45.3	40	45.4	0.950
Disagree		28	21	7	7	8.0	-
Not sure		184	143	47.7	41	46.6	-
**Information leaflet is sufficient to provide info on the procedure (*N* = 380)**							
Agree	16	188	143	49.1	45	50.6	0.973
Disagree		26	20	6.9	6	6.7	-
Not sure		166	128	44.0	38	42.7	-
**Wiling to test with partner using HIVST kit (*N* = 385)**							
Yes	11	325	255	86.1	70	78.7	0.185
No		35	25	8.5	10	11.2	-
Not sure		25	16	5.4	9	10.1	-

*Source:* Mokgatle, M. & Madiba, S., 2017, ‘High acceptability of HIV self-testing among technical vocational education and training college students in Gauteng and North West Province: What are the implications for the scale up in South Africa?’, *PLoS One* 12(1), 1–14. https://doi.org/10.1371/journal.pone.0169765

HIVST, HIV self-testing.

To determine the factors associated with the use and acceptability of HIVST among students in the TVET college, logistic regression analysis was performed (Table 4). Independent variables included having a sexual partner, number of sexual partners, condom use during last sexual encounter, ever tested for HIV, HIVST with partner, level of study, gender, knowledge of partner’s status and willingness to submit HIVST results. The researchers began by using computed bivariate analysis, followed by logistic regression to confirm the association. The outcome variable for the analysis was the acceptability of HIVST. The logistic regression showed that students who were willing to take up HIVST were at most ten times more likely to test using HIVST with their partners at unadjusted odds ratio (odds ratio [OR]: 10.23; 95% confidence interval [CI]: 5.49–19.07).

**TABLE 4:** Sexual behaviours related to the use and acceptability of HIV self-testing.

Variables	Yes		No		*N*	*p*	Unadjusted OR (95% CI)	Adjusted OR (95% CI)
	*n*	%	*n*	%				
**Sexually active**					367	0.000	1.231	2.142–6.943
Yes	52	17.39	247	82.61				
No	14	20.59	54	79.41				
**Number of sexual partners**					367	0.000	1.045	1.982–10.020
One	41	17.67	191	82.33				
Two or more	23	17.04	112	82.96				
**Condom use during the last sexual encounter**					367	0.000	0.6218	3.811–9.596
Yes	46	21.00	173	79.00				
No	21	14.19	127	85.81				
**HIVST with partner**					382	0.000	10.238	5.495–19.077
Yes	37	11.42	287	88.58				
No	33	56.90	25	43.10				
**Gender**					381	0.000	1.306	2.185–6.034
Male	19	21.59	69	78.41				
Female	51	17.41	242	82.59				
**Knowledge of partner’s status**					357	0.000	0.7094	3.573–9.705
Yes	45	19.31	188	80.69				
No	18	14.52	106	85.48				

*Source:* Mokgatle, M. & Madiba, S., 2017, ‘High acceptability of HIV self-testing among technical vocational education and training college students in Gauteng and North West Province: What are the implications for the scale up in South Africa?’, *PLoS One* 12(1), 1–14. https://doi.org/10.1371/journal.pone.0169765

OR, odds ratio; CI, confidence interval; HIVST, HIV self-testing.

## Discussion

The WHO has recommended HIVST as a safe and accurate testing modality to reach people who are least likely to test, including young people, and its promise, benefits and high acceptability have been documented globally (Figueroa et al. 2015). Moreover, SA incorporated the HIVST modality as a complementary strategy in the HIV Testing Services Policy to expand access to HIV testing services to the hard-to-reach populations such as young people who are least likely to utilise health facility–based HIV testing (Venter et al. 2017). It is against this backdrop that the present study assessed the students’ sexual behaviours and acceptability and perceived use of HIVST in a predominantly urban area at a TVET college. It was found that 36% of the students in this study reported having multiple sexual partners. This result matches the findings of Izigzag et al. (2018) in the DRC. It was also found that 40% of the students who were sexually active did not use a condom in their last sexual act. This result is similar (48%) to the DRC study of the university students in Kikwit University (Izigzag et al. 2018). The result was lower in comparison to the findings of Mokgatle and Madiba (2017) (66.5%) among TVET college students in South Africa. This may be owed to the context of the study setting and the study sample.

The study showed that there were no significant gender differences with regard to the acceptability of HIVST (*p* = 0.45). The data suggest that the acceptability of HIVST is high among students of Capricorn TVET College. These findings are consistent with other studies in sub-Saharan Africa (Choko et al. 2011; Izigzag et al. 2018; Kalibala et al. 2014; Pant Pai et al. 2014; Ritchwood et al. 2019). A randomised control study in SA among young women aged 18–26 years also found that an overwhelming majority of the study participants chose HIVST over HIV counselling and testing at a government clinic (Pettifor et al. 2020). The high HIVST acceptability among the students shows more substantial evidence that this innovative testing modality could increase HIV testing among the students in institutions of higher learning. In terms of the willingness to test with a partner, the majority reported their intention to test with their sexual partner. In a study among South African young adults, the study participants reported the preference to complete the test in the presence of significant others such as a relative, friend or sexual partner (Ritchwood et al. 2019). Izizag et al. (2018) also found that one of the factors associated with HIVST was the willingness to take up the test with a partner. It is feasible that scaling up the HIVST could increase reach, not only to the end users but also to significant others such as intimate partners, family and peers through secondary distribution. This shows that giving persons a choice to test in the comfort of their own space can be empowering. This further highlights the need for HIVST marketing in institutions of higher learning in SA. Although HIVST may be an attractive strategy to reach those who would not access the traditional methods, individuals still require linkages to health facilities to confirm the HIV diagnosis, treatment, care and support. A study by Sithole et al. (2022) investigating secondary distribution of HIVST kits in SA also confirmed that HIVST can get more people to test but emphasises that more support is needed to ensure successful linkage to care.

In the present study, it was also found that over three-quarters (78.9% female, 73% male) of the students were willing to confirm a HIVST positive test result at a local health facility. In a similar study in SA, the authors also found that over three-quarters (75%) reported their intention to confirm their HIV-positive result at a local clinic (Mokgatle & Madiba 2017). However, this result was higher than that of Izizag et al. in the DRC (66%) and Kurth et al. in Kenya (35.5%). Although HIVST offers promise to be one of the novel modalities to young people – *the worried well* – it is critical to highlight the health system structural barriers that need to be addressed to ensure that students are connected to confirm a positive test. These critical barriers (access, convenience, stigma, confidentiality, long waiting times and control over disclosing one’s status) have been cited in a number of studies (Kurth et al. 2016; Lippman et al. 2016; Madiba et al. 2015; Pai et al. 2013).

In other studies, it was argued that pre- and post-test counselling remains a bottleneck to scale up HIVST (Izigzag et al. 2018; Makusha et al. 2015). This is true of the findings in this study, as less than half of the students revealed that telephone hotlines are sufficient for post-test counselling. Similarly, Choko et al. (2011) found that the majority of the study participants reported that telephone hotlines were not a replacement for face-to-face counselling. Psychological risks following a positive HIVST result have been argued against (Mavedzenge, Baggaley & Corbett 2013). Similarly, the study by Ritchwood et al. (2019) reported that study participants were anxious that a positive HIVST result may lead to irrational behaviour and suicide attempts. Contrary to Mavedzenge et al. and Ritchwood et al., it has been disputed that arguments against HIVST were largely based on fear (Richter et al. 2012). Furthermore, a review by Brown et al. concluded that there was no evidence of harm following an HIVST positive result (Brown et al. 2014). There is no doubt that HIVST is an innovative strategy that can make testing accessible to students in institutions of higher learning; however, strategies for quality pre- and post-test counselling should be developed.

The willingness to take up HIVST with a partner was an important factor associated with the acceptability of HIVST in this study. This result matches the findings of Izigzag et al. (2018). This innovative and flexible strategy creates the potential to reach men in comparison to other traditional methods such as facility-based HIV testing. Similarly, it can be empowering for young women to initiate couple testing without evoking tensions from their male counterparts (Kumwenda et al. 2014).

## Recommendations and conclusion

A high number of students in this study reported HIVST as an acceptable HIV testing modality. The results suggest that successful implementation of HIVST including secondary distribution is feasible in institutions of higher learning in SA. Additionally, innovative demand creation for HIVST is critical in sexual reproductive health programming. Programme designers could leverage social media digital platforms such as Facebook, TikTok and Twitter to reach this high-priority population. Furthermore, the Ministry of Health should consider strategies to strengthen linkages or referral systems for HIVST confirmation and continuum of care. Future research should pursue the successful linkages to care for those who need to confirm an HIVST positive test.
